# Did the Physical and Psychological States of Outpatients Receiving Rehabilitation at a Geriatric Health Services Facility Decline during the State of Emergency Caused by the COVID-19 Pandemic?

**DOI:** 10.3390/diseases8040045

**Published:** 2020-12-10

**Authors:** Kazuhiro P. Izawa, Masataka Oyama, Keisuke Okamoto

**Affiliations:** 1 Department of Public Health, Graduate School of Health Sciences, Kobe University, 10-2 Tomogaoka 7-chome, Suma-ku, Kobe 654-0142, Japan; 2 Cardiovascular Stroke Renal Project (CRP), Kobe 654-0142, Japan; 3 Geriatric Health Services Facility Elder Village, 882 Fukutani, Hasetani-cho, Nishi-ku, Kobe-shi, Hyogo 651-2233, Japan; masataka.oyama@asunaro-grp.jp (M.O.); keisuke.okamoto@asunaro-grp.jp (K.O.)

**Keywords:** Coronavirus disease 2019, pandemic, Geriatric Health Services Facilities, outpatients, rehabilitation, physical state, psychological state

## Abstract

Many Geriatric Health Services Facilities in Japan may have continued outpatient rehabilitation by taking measures against infection even during the state of emergency caused by Coronavirus disease 2019 (COVID-19). The present study aimed to determine differences in physical and psychological states in rehabilitation outpatients (age, 83.5 ± 8.4 years) at a Geriatric Health Services Facility between the pre- and post-nationwide state of emergency in Japan. Physical outcomes were assessed with gait speed (GS), timed up and go test (TUG), handgrip strength (HG), and maximum phonation time (MPT). We used the Japanese version of the five-level EuroQoL five-dimensional questionnaire (EQ-5D-5L) to assess patients’ quality of life (QoL) as the psychological state. The physical (GS, pre, 0.92, post, 0.92 s, *p* = 0.875; TUG, pre, 14.09, post, 14.14 s, *p* = 0.552; HG, pre, 19.42, post 19.70 kgf, *p* = 0.807; MPT, pre, 13.6, post, 13.8 s, *p* = 0.861) and psychological (EQ-5D-5L, pre, 0.73, post, 0.81, *p* = 0.064) states of the participants did not change significantly between the pre- and post-nationwide state of emergency. This was likely due to the continuance of outpatient rehabilitation in accordance with the facility’s policy while taking adequate safety precautions against COVID-19 infection.

## 1. Introduction

The number of populations infected by Coronavirus disease 2019 (COVID-19) has been increasing worldwide [1]. The World Health Organization declared COVID-19 a pandemic on 11 March 2020 [2]. The government in Japan declared a state of emergency on 7 April 2020, and lifted it on 25 May 2020 [3]. These policies played a role in inhibiting the expansion of the outbreak of infection [4], but health problems affected by restrictions on activity are a concern.

It was previously reported that physical activity (PA) was significantly decreased due to the COVID-19 epidemic in community-dwelling elderly Japanese adults [5]. That study performed a subgroup analysis according to frailty categories, and PA was significantly decreased among the subjects in all categories [5]. However, quite a few Geriatric Health Services Facilities may have continued outpatient rehabilitation by taking measures against infection even during the state of emergency. Thus, we hypothesized that the physical and psychological states would decrease in people receiving outpatient rehabilitation in Geriatric Health Services Facilities under the influence of the emergency restrictions.

The purposes of the present study were to determine the differences in physical and psychological states of outpatients receiving rehabilitation at a Geriatric Health Services Facility between the pre- and post-nationwide state of emergency in Japan.

## 2. Materials and Methods

This was a longitudinal study of 20 consecutive elderly participants who received outpatient rehabilitation at the Geriatric Health Services Facility Elder Village in Kobe, Japan, from January 2020 to the end of May 2020. We excluded participants from the present study who did not give informed consent, could not walk by themselves, and who could not attend outpatient rehabilitation during the state of emergency due to the desire and/or personal convenience of the participant or the participant’s family. The intervention of outpatient rehabilitation at the Geriatric Health Services Facility was performed once or twice a week (20 min/session) during the state of emergency. Each session included a warm-up period, resistance training, aerobic exercise, and a cooldown period. In the first session, outpatients performed a series of upper and lower limb and body stretches before and after the exercise. Exercise intensity was performed to maintain heart rate at a rating of perceived exertion of 11–13 on the Borg scale as based on a previous method [6].

At that time, infection prevention measures were strictly implemented for the participants and staff, which included wearing masks, washing hands, and disinfecting equipment.

Characteristics of the participants’ data were evaluated from the patients’ medical records and included age, sex, body mass index (BMI), long-term care insurance level, living alone, diagnosis, and medications.

Physical outcomes were assessed with gait speed [7,8], the timed up and go test [7], handgrip strength [8], and maximum phonation time [9], based on previous methods. We used the Japanese version of the five-level EuroQoL five-dimensional questionnaire (EQ-5D-5L) to assess the patients’ quality of life (QoL) [10,11] as their psychological state. The EQ-5D-5L is a reliable and sensitive assessment in Japanese patients [10,11]. The EQ-5D-5L is a self-reported questionnaire in which patients report their own evaluations of their current health state [12]. The EQ-5D-5L consists of five items: mobility, self-care, usual activities, pain/discomfort, and anxiety/depression, and each item has five levels of description [12]. The QoL score is calculated by a value set determined beforehand to reflect the preferences of the general population. The EQ-5D-5L QoL score ranges from 0 (death) to 1 (full health). The physical and psychological states were assessed by physical and occupational therapists at two time points: before and after the nationwide state of emergency in Japan. We complied with the principles of the 1975 Declaration of Helsinki regarding investigations in human subjects, and we obtained informed consent from each participant.

Characteristics of the participants and evaluated results are shown as numbers and as the mean ± standard deviation for the continuous variables. We analyzed longitudinal changes of the physical and psychological states from January, 2020 (pre-nationwide state of emergency) to the end of May, 2020 (post-nationwide state of emergency) using the Wilcoxon signed-rank test. The overall level of statistical significance was set at 0.05. Statistical analyses were performed with IBM SPSS Statistics 26 (IBM SPSS, Tokyo, Japan).

## 3. Results

### 3.1. Characteristics of the Participants

Of the 20 patients, 5 were excluded because they could not attend outpatient rehabilitation during the state of emergency due to the desire and/or personal convenience of the participant or the participant’s family, and 2 patients could not walk by themselves. Thus, the final analysis comprised 13 patients (age, 83.5 ± 8.4 years; BMI, 24.6 ± 5.5 kg/m^2^; male, 6/13; long-term care insurance support level 2, 9/13, care level 1, 2/13, and care level 2, 2/13; and living alone, 2/13). Patient diagnoses were orthopedic disease in 7, internal disease in 4, and cerebrovascular disease in 2 patients. Main medications taken were angiotensin II receptor blocker in 3, β-blocker in 3, calcium antagonist in 7, diuretic in 3, statin in 5, and analgesics in 5 patients.

### 3.2. Results of Physical and Psychological Tests

The results of the physical and psychological testing of the participants did not change significantly between January, 2020 (pre-nationwide state of emergency) and the end of May, 2020 (post-nationwide state of emergency) (*p* < 0.01) (Table 1).

## 4. Discussion

To the best of our knowledge, this is the first study to investigate differences in physical and physiological states of outpatients undergoing rehabilitation at a Geriatric Health Services Facility between the pre- and post-nationwide state of emergency in Japan. We showed that neither physical nor psychological states had decreased significantly between the two time points (Table 1). In other words, both the physical and physiological states of the participants could be maintained during this period.

A previous study reported that PA significantly decreased due to the COVID-19 epidemic in community-dwelling older adults in Japan [5]. Moreover, it was reported that stress, depression, anxiety, PA, and QoL worsened due to the COVID-19 epidemic in patients with Parkinson’s disease [13]. Our results suggested a tendency dissimilar to those of this other preliminary research [5,13] and that the policies enacted during the state of emergency in Japan might not affect either state in rehabilitation outpatients.

In terms of long-term care providers, in response to the state of emergency for COVID-19, the basic coping policy of the Geriatric Health Services Facility in Japan states that “businesses are required to continue their business when an emergency is declared” [14]. Even during the state of emergency, this organization continued in accordance with its facility policy. It is possible that this contributed to the maintenance of patient states during the study period.

There are several limitations in the present study. First, this study investigated a single Geriatric Health Services Facility, and the sample size was very small; thus, the generalizability of the results may be limited. Second, there was no control group, so we do not know the outcome of the Geriatric Health Services Facility patients who did not participate in outpatient rehabilitation during the state of emergency. Finally, there were no data on different outcomes due to different outpatient rehabilitation frequencies. Thus, further studies are needed to clarify the long-term effects of the COVID-19 epidemic on the relationship between physical and psychological states in these patients.

## 5. Conclusions

In conclusion, our results showed that the policies related to the state of emergency in Japan did not appear to have affected the physical and psychological states of elderly Japanese patients participating in outpatient rehabilitation. One reason may be that outpatient rehabilitation continued in accordance with the facility’s policy even during the state of emergency while taking adequate safety precautions against COVID-19 infection.

## Figures and Tables

**Table 1:** Differences in results of physical and psychological testing between the pre- and post-nationwide state of emergency.

Tests	Pre-Nationwide State of Emergency	Post-Nationwide State of Emergency	Standardized Test Statistic	*p* Value
Physical				
GS (m/s)	0.92 ± 0.33	0.92 ± 0.34	−0.157	0.875
TUG (s)	14.09 ± 6.15	14.14 ± 6.56	0.594	0.552
HG (kgf)	19.42 ± 5.85	19.70 ± 7.07	0.245	0.807
MPT (sec)	13.6 ± 4.83	13.8 ± 3.28	0.175	0.861
Psychosocial				
EQ-5D-5L QoL scores	0.73 ± 0.11	0.81 ± 0.15	1.852	0.064

Data are presented as mean ± standard deviation. GS, gait speed; TUG, Timed Up and Go test; HG, handgrip strength; MPT, Maximum Phonation Time; EQ-5D-5L, five-level version of the EuroQoL five-dimensional questionnaire; QoL, Quality of Life.
